# Efficacy and Safety of Oral and IV Levonadifloxacin Therapy in Management of Bacterial Infections: Findings of a Prospective, Observational, Multi-center, Post-marketing Surveillance Study

**DOI:** 10.7759/cureus.55178

**Published:** 2024-02-28

**Authors:** Sanjith Saseedharan, Kapil Zirpe, Yatin Mehta, Dilip Dubey, Anand Sutar, Khokan Debnath, Sanket Newale

**Affiliations:** 1 Critical Care, SL Raheja Hospitals - A Fortis Associate, Mumbai, IND; 2 Neurocritical Care, Ruby Hall Clinic, Grant Medical Foundation, Pune, IND; 3 Medanta Institute of Critical Care and Anesthesiology, Medanta - The Medicity, Gurugram, IND; 4 Pulmonology, Medanta Hospital, Lucknow, IND; 5 Critical Care Medicine, Apollo Hospitals, Bengaluru, IND; 6 Clinical Operations, Regulatory Affairs, Pharmacovigilance and Quality Assurance, Wockhardt Ltd., Mumbai, IND; 7 Medical Affairs, Wockhardt Ltd., Mumbai, IND

**Keywords:** bacterial infection, “methicillin-resistant staphylococcus aureus”, microbial success, clinical success, levonadifloxacin, covid-19

## Abstract

Background

Antimicrobial resistance by bacteria poses a substantial threat to morbidity and mortality worldwide, and treatment of resistant infections is a challenge for the treating clinician. Levonadifloxacin is a novel broad-spectrum agent belonging to the benzoquinolizine subclass of quinolone, which can be used by both oral and intravenous administration for the treatment of infections caused by gram-positive organisms, including methicillin-resistant Staphylococcus aureus (MRSA).

Patients and methods

This prescription event monitoring study captured data from 1266 patients receiving levonadifloxacin (oral and/or IV) in a real-world setting to assess the safety and efficacy in the treatment of various bacterial infections. The duration of the study was 18 months. Study outcomes were clinical success and microbial success at the end of therapy. Global assessments were done for safety and efficacy at the end of therapy using a 5-point Likert scale (excellent, very good, good, satisfactory, and poor).

Results

The mean (median) duration of therapy was 7.2 (7.0) days, with a median time to clinical improvement of four days. Oral therapy was administered to 224 patients; 940 received IV, and 102 received IV followed by oral therapy. Patients were prescribed levonadifloxacin for gram-positive infections, skin and soft tissue infections, diabetic foot infections, septicemia, catheter-related blood-stream infections, bone and joint infections, febrile neutropenia, and respiratory infections, including COVID-19 pneumonia. The clinical cure on the eighth day was 95.7%, whereas the microbial success on the eighth day was 93.3% (n=60). For different types of infections, the clinical success rates ranged from 85.2% to 100%. There were only 30 treatment-emergent adverse events reported in 29 patients. Overall, about 95.6% of patients rated the efficacy as good to excellent, whereas only 3.8% of patients rated it satisfactory; for safety, 95.7% of patients rated it as good to excellent, with only 3.9% of patients rated it as satisfactory.

Conclusions

The excellent safety and efficacy profile of levonadifloxacin, when administered as an oral or intravenous therapy, makes it a desirable treatment modality for the management of various bacterial infections, including those caused by resistant pathogens such as MRSA and quinolone-resistant Staphylococcus aureus (QRSA). Features of levonadifloxacin, such as availability in both IV and oral form, minimal drug-drug interactions, lack of the need to adjust dosages in renal and hepatically impaired patients along with a broad spectrum of coverage, make it a suitable agent that meets several unmet clinical needs of physicians.

## Introduction

The spread of antibiotic-resistant bacteria poses a substantial threat to morbidity and mortality worldwide. Gram-positive organisms (including bacteria of the genera Staphylococcus, Streptococcus, and Enterococcus) are among the most common bacterial causes of clinical infection. This is primarily due to their association with a diverse spectrum of pathology, ranging from mild skin and soft tissue infections to life-threatening systemic sepsis and meningitis [1]. Although recent global attention has focused on the serious issues associated with multiple drug-resistant (MDR) gram-negative infections, even MDR gram-positive infections are causing serious health concerns. Methicillin-resistant Staphylococcus aureus (MRSA) is perhaps the paradigm example and is of high global importance as a cause of community-acquired and healthcare-associated infection [2]. The rate of MRSA bacteremia in Canada, Australia, and Scandinavia increased from 2000 to 2008 due to a rise in community-acquired infections [3,4]. In India, high rates of MRSA have been reported in clinical isolates from various studies, with rates as high as 54.8% (ranging between 32% and 80% among the S. aureus pool) [5]. According to the study conducted by the Indian Council of Medical Research (ICMR), the prevalence of MRSA in India was 37.3% in 2015, which rose to 38.6% in 2018. In a recent ICMR Antimicrobial Resistance Surveillance and Research Network (AMRSN) report, North India demonstrated elevated MRSA rates (52.8%), followed by West (48.1%) and East India (42.5%) [6]. Resistant bacterial infections, including MRSA and methicillin-susceptible Staphylococcus aureus (MSSA), are not only major health issues but also major causes of financial burdens all over the world.

Levonadifloxacin is approved for use in bacterial infections and is available in India as both oral and injectable formulations. It is found to be highly active against gram-positive organisms like Staphylococcus aureus (methicillin-resistant, methicillin-susceptible, quinolone-resistant, quinolone-susceptible isolates), Streptococcus pyogenes, Enterococcus faecalis, Streptococcus dysgalactiae. Levonadifloxacin has the potential to be useful against respiratory tract infections caused by organisms such as Streptococcus pneumoniae, Haemophilus influenzae, Moraxella catarrhalis, quinolone-susceptible gram-negative bacteria, and atypical bacteria. Due to higher intracellular concentrations in different tissues, levonadifloxacin is a better-suited antimicrobial agent (AMA) for the treatment of skin and soft tissue infections caused by both gram-positive and gram-negative bacteria [7]. Also, levonadifloxacin has an excellent safety profile, which makes it a better option for the treatment of various bacterial infections.

This study presents the results of levonadifloxacin oral and/or injection therapy administered to treat various bacterial infections in a real-world setting.

## Materials and methods

Setting

This data is part of a multicenter, prospective, post-marketing, observational study conducted for the assessment of the efficacy and safety of levonadifloxacin in various bacterial infections in a real-world setting. Results of levonadifloxacin therapy administered as oral/injection in bacterial infections are included from 76 sites/hospitals across India reported in this study. The duration of the study was 18 months.

Informed consent and ethics

As a part of the post-marketing observational study, data from 1266 patients who received levonadifloxacin therapy in any form for various bacterial infections was collected from participating sites. The study documents were reviewed and approved by the Institutional Ethics Committee (IEC) of DY Patil University School of Medicine, Navi Mumbai (DYP/IEC/18/2021, July 08, 2021). The study was conducted in accordance with the principles of the Declaration of Helsinki (World Medical Association) and Good Clinical Practice (GCP) guidelines issued by the ICMR and The Central Drugs Standard Control Organisation (CDSCO), Government of India, and was registered with the clinical trials registry of India (CTRI/2021/08/035789, August 17, 2021). This being a prospective study, patient consent was obtained from all patients before any data collection, and strict confidentiality was maintained for the patients' identities.

Study participants

Data from 1266 patients of any gender above 18 years of age who were prescribed levonadifloxacin (oral or injectable) was included in the study. The diagnosis of bacterial infections was based on either clinical judgment or microbial test results. Data was collected and recorded in a study-specific data capture web-based tool from 76 participating sites. Patient information like demography, their clinical condition on admission, comorbidities, complications, and details of other treatments (including antimicrobial agents) received by them was collected. Also, information about hospitalization and the duration of levonadifloxacin therapy was collected. Microbial testing data was collected wherever available. This being an observational study, there were no study-specific treatments, and all treatments for the patients were based at the discretion of the treating clinician.

Study treatments

Levonadifloxacin IV injection and oral levonadifloxacin tablet, i.e., EMROK® (levonadifloxacin injection 800mg/100ml) and EMROK-O® (levonadifloxacin tablet 500 mg) as prescribed by their treating physicians.

Study outcomes

The study outcomes were clinical response and microbial response at the end of therapy. Clinical response was assessed as clinical success (complete disappearance of clinical signs and symptoms within the treatment period), clinical improvement (subsidence of clinical signs and symptoms but with incomplete resolution), or clinical failure (unchanged or worsening of clinical signs and symptoms present at baseline). Clinical failure also included the need for additional antimicrobial agents and the occurrence of new infections or death. Microbial response was assessed as microbial success (absence of baseline pathogen in post-treatment microbial evaluation) or microbial failure (persistence of baseline pathogen in post-treatment microbial evaluation).

Overall global assessment for efficacy was determined by the treating clinician based on a 5-point Likert scale (excellent, very good, good, satisfactory, and poor). The safety of treatments received by the patients was assessed using the documented clinical adverse events and laboratory parameters. Overall global assessment for safety was done by the treating clinician for each patient based on a 5-point Likert scale (excellent, very good, good, satisfactory, and poor).

Statistical analysis

This being an observational study, there is no study hypothesis, and no statistical testing was done. The data were entered in MS Office Excel (Microsoft Corp., Redmond, US). Descriptive statistics are presented for demography and study outcomes. Measurement data are presented as means and standard deviation (SD), whereas categorical data is presented as numbers with percentages. 

## Results

Of the 1266 patients included in the final analysis, there were 851 (67.2%) males and 415 (32.8%) females. Patients had a median age of 55.5 years (ranging from 18 to 93 years). Table 1 presents the demography, treatment setting (outpatient or hospitalized), presence of comorbid conditions, and duration of levonadifloxacin therapy in the patients. The most common comorbid conditions reported in patients were diabetes (12.5%) and hypertension (3.2%). Other comorbidities were renal disorders (1.7%), ischemic heart disease, respiratory disorders, thyroid disorders, hepatic disorders, and malignancy.

**Table 1:** Profile of patients and levonadifloxacin therapy received by the patients (n=1266)

Characteristics	No. (%)	Mean (SD)	Median (range)
Age (years)	-	57.10 (14.46)	55.50 (18.00-93.00)
BMI (kg/m^2^)	-	25.77 (4.123)	39.54 (14.45-64.64)
Gender			
Male	851 (67.2%)	-	-
Female	415 (32.8%)	-	-
Treatment setting			
Outpatient	237 (18.7%)	-	-
Hospitalized	1029 (81.3%)	-	-
Comorbid conditions			
No comorbidity	961 (75.9%)	-	-
One comorbidity	285 (22.5%)	-	-
Two comorbidities	18 (1.4%)	-	-
Three comorbidities	2 (0.2%)	-	-
Levonadifloxacin therapy (days)			
IV therapy	940 (74.2%)	6.96 (3.15)	16.00 (1.00-31.00)
Oral therapy	224 (17.7%)	7.58 (3.57)	15.50 (3.00-28.00)
IV followed by oral therapy	102 (8.1%)	14.31 (7.77)	27.00 (5.00-49.00)

Most of the patients (n=940, 74.2%) received only IV levonadifloxacin, whereas 224 (17.7%) patients received only oral therapy, and 102 (8.1%) received IV followed by the switchover to oral therapy. Levonadifloxacin was prescribed as empirical therapy in 1107 (87.4%) patients. It was prescribed as monotherapy in 407 (32.1%) patients. Of the 1266 patients, 1029 (81.3%) patients were hospitalized, whereas 237 (18.7%) patients were treated on an outpatient basis. Table 2 presents the body systems involved in bacterial infection and existing complications in the patients who were prescribed levonadifloxacin. Different complications of infections were reported in patients with renal impairment (16.4%) and septic shock (21.8%) as the common complications. Systemic inflammatory response syndrome (SIRS) was reported in 7.8% of patients. Other complications included multiorgan failure, hepatic impairment, and thrombocytopenia.

**Table 2:** Systems involved and complications of infection SIRS - systemic inflammatory response syndrome

System involved in infection	No.	%
Abdominal	184	14.5%
Pulmonary	580	45.8%
Cardiovascular	51	4.0%
Skin / soft tissue	288	22.7%
Pelvic	19	1.5%
Neurological/meningeal	36	2.8%
Retroperitoneal	10	0.8%
Other systems Involved	193	15.2%
Complication of infection		
Septic shock	276	21.8%
Renal impairment	207	16.4%
SIRS	99	7.8%
Multiorgan failure	96	7.6%
Other complications	88	7.0%
Thrombocytopenia	79	6.2%
Hepatic Impairment	52	4.1%

Table 3 depicts the results of microbial evaluation at baseline. Gram-positive organisms were isolated from 417 patients, gram-negative organisms were isolated from 66 patients, and atypical organisms were isolated from six patients. Organisms isolated were S. aureus (MRSA and MSSA), Streptococci, Klebsiella, Salmonella, Enterococci, Pseudomonas, Escherichia coli, Acinetobacter spp., vancomycin-resistant Enterococcus faecium, Serratia marcescens, and Stenotrophomonas maltophilia. The concomitant antimicrobial agents used in the patients receiving levonadifloxacin were carbapenems and beta-lactam antibiotics.

**Table 3:** Bacteria isolated at baseline

Bacteria	No.	%
Gram-positive organisms (n=417)		
Staphylococcus aureus	302	72.50%
Streptococcus pneumoniae	38	9.10%
Enterococcus faecalis	27	6.50%
Staphylococcus epidermidis	16	3.80%
Vancomycin-resistant Enterococcus	9	2.20%
Acinetobacter	6	1.40%
Coagulase-negative Staphylococcus	4	1.00%
Enterococcus faecium	4	1.00%
Beta-haemolytic streptococci	2	0.50%
Staphylococcus intermedius	2	0.50%
Streptococci pyogenes	2	0.50%
Clostridium Ttetani	1	0.20%
Micrococcus species	1	0.20%
Staphylococcus haemolyticus	1	0.20%
Staphylococcus hominis	1	0.20%
Staphylococcus saprophyticus	1	0.20%
Gram-negative organisms (n=66)		
Enterobacteriaceae	24	36.90%
Moraxella catarrhalis	5	7.70%
Proteus mirabilis	5	7.70%
Citrobacter species	2	3.10%
Flavobacterium	2	3.10%
Actinomyces	2	3.10%
Burkholderia mallei	1	1.50%
Chryseobacterium	1	1.50%
Acinetobacter baumannii	1	1.50%
Other	23	35.40%
Atypical organisms (n=6)		
Mycoplasma pneumoniae	4	66.70%
Legionella pneumophila	1	16.70%
Other	1	16.70%

Levofloxacin was used after the failure of a previous antimicrobial agent in 234 (18.5%) patients. Table 4 shows the most common antimicrobials used prior to levonadifloxacin, which were macrolides, beta-lactam antibiotics (penicillin, cephalosporins, and carbapenems), glycopeptide antibiotics, and fluoroquinolones.

**Table 4:** Antimicrobials used in patients

Drug	No.	%
Azithromycin	35	2.8%
Meropenem	28	2.2%
Vancomycin	19	1.5%
Amoxicillin clavulanic	15	1.2%
Cefoperazone sulbactam	15	1.2%
Clarithromycin	13	1.0%
Teicoplanin	13	1.0%
Piperacillin tazobactam	12	0.9%
Clindamycin	11	0.9%
Ampicillin sulbactam	10	0.8%
Linezolid	8	0.6%
Levofloxacin	7	0.6%
Amikacin	5	0.4%
Polymyxin B	5	0.4%
Cefoperazone	4	0.3%
Ceftriaxone	4	0.3%
Doxycycline	4	0.3%
Cefazolin	3	0.2%
Cefepime	3	0.2%
Cefpodoxime	3	0.2%
Tigecycline	3	0.2%
Cefixime	2	0.2%
Colistin	2	0.2%
Aztreonam	1	0.1%
Ceftazidime	1	0.1%
Gentamicin	1	0.1%
Imipenem	1	0.1%
Minocycline	1	0.1%
Netilmicin	1	0.1%
Fosfomycin	1	0.1%
Others	3	0.2%

Table 5 shows the different indications for the use of levonadifloxacin. Lower respiratory tract infections (37.5%), acute bacterial skin and soft tissue infections (21.2%), and septicemia (18.9%) were the most common indications for the use of levonadifloxacin. Other indications were febrile neutropenia (4.7%), device-related infections (4.5%), urinary tract infection (3.6%), bone and joint infections (2.1%) and abdominal infections (1.7%).

**Table 5:** Indications for levonadifloxacin therapy (n=1266) ABSSSI - acute bacterial skin and skin structure infections; LRTI - lower respiratory tract infections; URTI - upper respiratory tract infections; BJI - bone and joint infection; UTI - urinary tract infection

Indication	No.	%
LRTI	475	37.5
ABSSSI	268	21.2
Septicaemia	239	18.9
Febrile neutropenia	59	4.7
Device-related infection	57	4.5
UTI	46	3.6
BJI	27	2.1
Intra-abdominal infection	21	1.7
URTI	12	0.9
Cardiac infection	11	0.9
Musculoskeletal/connective tissue infection	9	0.7
Others	42	3.3

Clinical and microbial success

Table 6 presents the clinical response on the third and eighth days, i.e., the end of levonadifloxacin therapy. Clinical cure was observed in 95.7% of patients at the end of therapy. Table 6 depicts the microbiological response on the eighth day based on the post-treatment culture, which was done in 60 patients. Thus, data from only 60 patients is available, where the microbial success rate was 93.3% (56/60) on the eighth day, with microbial failure occurring in only four patients (6.7%).

**Table 6:** Clinical and microbial response to levonadifloxacin therapy EOT - end of treatment

Clinical and microbial response	Day 3		Day 8 (EOT)	
	No.	%	No.	%
Clinical response (n=1266)				
Clinical cure	946	74.7%	937	95.7%
Clinical failure	320	25.3%	42	4.3%
Microbial response (n=60)				
Microbiological success	-	-	56	93.3%
Microbiological failure	-	-	4	6.7%

Table 7 shows the clinical and microbial success rates in different sub-groups on the eighth day.

**Table 7:** Clinical and microbial success rates (%) in different sub-groups on the eighth day ABSSSI - acute bacterial skin and skin structure infections; AMA - antimicrobial agents; ICU - intensive care unit; IV - intravenous; LRTI - lower respiratory tract infections; URTI - upper respiratory tract infections; BJI - bone and joint infection; UTI - urinary tract infection; N - total number of patients in sub-group; No. - total number of patients with the response on the eighth day

Sub-groups		Clinical success			Microbiological success		
		N	No.	%	N	No.	%
Gender	Male	651	621	95.4%	40	36	90.0%
	Female	328	316	96.3%	20	20	100.0%
Comorbidity	No comorbidity	718	689	96.0%	32	29	90.6%
	One comorbidity	241	228	94.6%	25	24	96.0%
	Two comorbidities	18	18	100.0%	2	2	100.0%
	Three comorbidities	2	2	100.0%	1	1	100.0%
Hospitalized	Not hospitalized	206	205	99.5%	4	4	100.0%
	ICU	569	543	95.4%	33	31	93.9%
	Ward	204	189	92.6%	23	21	91.3%
	Hospitalized (ICU + ward)	773	732	94.7%	56	52	92.9%
Levonadifloxacin started as	Empirical therapy	899	861	95.8%	41	37	90.2%
	After culture sensitivity	80	76	95.0%	19	19	100.0%
Levonadifloxacin indication	ABSSSI	240	234	97.5%	13	11	84.6%
	Musculoskeletal infection	9	9	100.0%	0	0	-
	LRTI	346	329	95.1%	18	17	94.4%
	URTI	10	10	100.0%	2	2	100.0%
	BJI	20	20	100.0%	0	0	-
	Device-related infection	43	42	97.7%	1	1	100.0%
	Septicemia	186	174	93.5%	12	11	91.7%
	UTI	33	33	100.0%	6	6	100.0%
	Intra-abdominal infection	18	17	94.4%	4	4	100.0%
	Febrile neutropenia	36	35	97.2%	3	3	100.0%
	Cardiac infection	11	11	100.0%	0	0	-
	Others	27	23	85.2%	1	1	100.0%
Reason for levonadifloxacin	First-line therapy	884	849	96.0%	47	44	93.6%
	Failure of other AMAs	95	88	92.6%	13	12	92.3%
Levonadifloxacin therapy type	Monotherapy	333	322	96.7%	15	14	93.3%
	Combination therapy	646	615	95.2%	45	42	93.3%
Levonadifloxacin therapy	IV therapy	684	645	94.3%	45	41	91.1%
	Oral therapy	205	204	99.5%	7	7	100.0%
	IV followed by oral therapy	90	88	97.8%	8	8	100.0%
	EMROK® IV only therapy	774	733	94.7%	53	49	92.5%
	EMROK® oral therapy	295	292	99.0%	15	15	100.0%
All patients	Total	979	937	95.7%	60	56	93.3%

Safety

Out of 1266 patients, there were only 30 treatment-emergent adverse events observed in 29 patients. The common adverse events reported were fever (n=5), constipation (n=4), diarrhea (n=4) and rashes (n=4). Most of the events were of mild to moderate severity and were successfully resolved.

Global assessments

**Figure 1:**
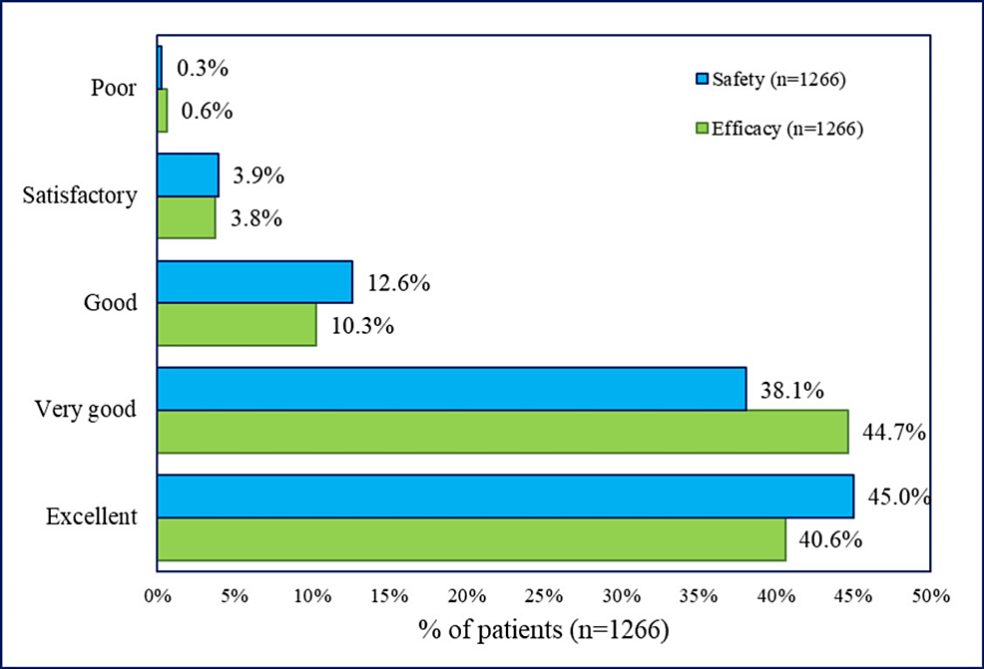
Global assessments for efficacy and safety of levonadifloxacin at the end of therapy

Figure 1 presents the global assessments for efficacy and safety at the end of therapy. Overall, about 95.6% of patients rated the efficacy as good to excellent, whereas only 3.8% of patients rated it as satisfactory efficacy, whereas 95.7% of patients rated the safety as good to excellent, with only 3.9% of patients rated it as satisfactory.

## Discussion

This prescription event monitoring study captured the real-world safety and efficacy data of the use of levonadifloxacin in the treatment of various bacterial infections. The data of 1266 patients were collected for the use of oral and IV levonadifloxacin from all regions of India, thus providing information about the actual benefit of levonadifloxacin in the treatment of bacterial infections. Patients were prescribed levonadifloxacin for varied indications like acute bacterial skin and soft tissue infection, bone and joint infection, catheter-related bloodstream infection, diabetic foot infection, respiratory tract infections, febrile neutropenia, COVID-19 pneumonia, and gram-positive infections.

Staphylococcus aureus, which is a facultative intracellular pathogen, is one of the common pathogens responsible for both community and hospital-acquired infections [8]. As reported in a meta-analysis by Ghia et al. in 2020, based on the results of four clinical studies, the treatment options identified for MRSA in India include arbekacin sulfate, vancomycin hydrochloride, teicoplanin, daptomycin and oritavancin [9]. However, the lack of intracellular activity of many of these anti-staphylococcal drugs could be responsible for persistent S. aureus infections, such as persistent bacteremia and failure of therapy. Levonadifloxacin and its ester oral prodrug, alalevonadifloxacin, are the broad-spectrum benzoquinolizine subclass of quinolones having activity against multi-drug-resistant gram-positive pathogens including MRSA, heterogeneous vancomycin-intermediate Staphylococcus aureus (hVISA), and vancomycin-resistant Staphylococcus aureus (VRSA), as well as quinolone-resistant strains [10]. Levonadifloxacin has been shown to have potent killing action against both the MSSA and MRSA in an intracellular environment of THP-1 macrophages [11,12]. The intracellular activity of levonadifloxacin was found to be better than that of other fluoroquinolones (moxifloxacin and ciprofloxacin) [13]. Thus, due to its high intracellular activity, both oral and IV levonadifloxacin can offer better therapeutic options for treating persistent MRSA infections. Levonadifloxacin has been shown to exhibit potent in-vitro activity against contemporary S. aureus isolates (MRSA isolates, hVISA isolates, Bengal Bay clone isolates, and quinolone-resistant isolates) collected from a large Indian tertiary care hospital, where 793 isolates were studied [14]. Levonadifloxacin showed MIC50 and MIC90 values of 0.25 and 0.5 mg/L, respectively, for all S. aureus strains. Levonadifloxacin has been shown to retain good potency against strains with multiple mutations in S. aureus in quinolone targets [15]. The majority of the patients in our study (66.2%) had gram-positive infections. With oral and IV levonadifloxacin, we observed clinical success rates of 96.1% against MRSA and 95.9% versus other gram-positive organisms. Additionally, a high systemic exposure along with higher attainment of intrapulmonary levels, as observed in epithelial lung fluid (ELF)and alveolar macrophage (AM), point toward the clinical utility of levonadifloxacin for the treatment of respiratory infections caused by extracellular and intracellular pathogens [10].

Our study included patients with mixed infections and possibly those with gram-negative bacterial infections. It has activity against gram-negative, atypical bacteria and anaerobes, and this offers a simplified mono-therapeutic advantage for the treatment of polymicrobial infections. Levonadifloxacin had the lowest MIC (0.5 mg/L and 2.0 mg/L) for anaerobic organisms (B. fragilis, Prevotella, Porphyromona, ß-lactamase-producing Fusobacteria, C. perfringens, and C. difficile) as compared to other fluoroquinolones [10]. It has been reported to have a potential role in the treatment of anaerobic and mixed bacterial infections [16]. Our study had 12.72% of patients (n=109) with polymicrobial infections, and the clinical success rates were more than 90.0% (data not presented). 

Febrile neutropenia is considered a medical emergency since the infection can rapidly progress, thus warranting immediate, highly effective broad-spectrum antibiotics [17]. Also, antibacterial prophylaxis with fluoroquinolone is recommended for all high-risk patients with neutropenia [18]. Immediate therapy is also indicated in patients with protracted neutropenia, like those with acute myeloid leukemia, myelodysplastic syndromes, or hematopoietic stem-cell transplantation treated with immunosuppressives [19]. The recommended daily doses of levofloxacin for preventing infections in neutropenia are 50 to 750 mg, and therapy should be started within 60 minutes of presentation in all patients presenting with neutropenic fever [17]. We had 36 patients with febrile neutropenia, of whom all except one responded (97.2% clinical success) to levonadifloxacin therapy.

Bone and joint infections include septic arthritis, prosthetic joint infections, osteomyelitis, spinal infections, and diabetic foot osteomyelitis. Early intervention with appropriate and targeted antimicrobial therapy is essential to resolve these infections. Foreign materials in the body provide an inert surface for microorganisms to adhere to, where they are relatively protected from the blood supply, immune processes, and antibiotics. Organisms such as Staphylococcus spp. are commonly involved in forming biofilms, which is a very important mechanism for bacterial survival in chronic bone and joint infection. The unique ability of levonadifloxacin to efficiently penetrate biofilms and exert its rapid bactericidal activity, even under acidic conditions, makes it a suitable agent for biofilm eradication. In addition, its broad spectrum of activity, paired with an excellent safety profile, makes levonadifloxacin a suitable antimicrobial agent for long-term therapy of bone and joint infections. Our study also included 27 patients with bone and joint infection, where all responded to levonadifloxacin therapy.

Results of the phase-3 study of levonadifloxacin versus linezolid in 500 patients with acute bacterial skin and skin structure infection (ABSSSI) have been recently published [20]. The clinical cure rates observed in the modified intent to treat (mITT) populations for levonadifloxacin were 91.0% with IV treatment and 95.2% with oral treatment. The authors conclude that both oral and IV levonadifloxacin are not inferior to linezolid for the treatment of ABSSSI. We observed a clinical success rate of 97.5% with levonadifloxacin therapy (oral/IV) for the treatment of ABSSSI. Table 7 shows that for all types of infections, we observed clinical success rates of 99.5% with oral therapy and 94.3% with IV therapy.

In our study, for most of the patients (95.6%), the global efficacy was reported as good to excellent. Multiple attributes of levonadifloxacin, such as not being a substrate for the NorA efflux pump, preferential DNA gyrase activity, and cidality against high-inoculum cultures and slow-growing staphylococci, contribute to resistance prevention. In addition, improved activity in acidic pH, activity against atypical bacteria, and potent activity against anaerobic isolates excluding B. fragilis would be of benefit in treating ABSSSI, diabetic foot, and bone and joint infection. We observed a clinical success rate of 98.8% in patients with diabetic foot infection.

The safety of levonadifloxacin is well documented. However, QT prolongation is a common risk associated with quinolones, and moxifloxacin is said to have the highest risk of QT prolongation among the available ones [21]. In a study involving 48 healthy subjects, a supratherapeutic dose (2600 mg of levonadifloxacin) was not found to be associated with any changes in baseline and placebo-corrected QTcF (QT interval corrected for heart rate by the Fridericia formula), QRS, or PR [22]. In all studies published to date, no deaths, serious adverse events, or significant abnormalities in clinical laboratory parameters, vital signs, 12-lead ECG changes, or findings on physical examination are being reported [23]. In our study, for most of the patients (95.7%), the global safety was reported as good to excellent.

Being devoid of potential adverse effects, such as phototoxicity, prolongation of QT interval, hepatotoxicity, and nephrotoxicity, levonadifloxacin can offer a valuable therapeutic option for the management of complex and serious bacterial infections. The excellent bioavailability of oral formulations in both the fasted and fed states is helpful in the smooth switch from parenteral to oral therapy [10]. The well-established pharmacokinetics and safety of both formulations of the drug show their potential for clinical use.

This study has some limitations in terms of being a prescription event monitoring study. Due to the observational study design, microbial testing results were not available/done for all patients before the start of therapy and after therapy. Hence, less data was available for microbial success, which limits the extrapolation of study results to large-scale populations.

## Conclusions

The excellent safety and efficacy profile of levonadifloxacin, when administered as an oral or intravenous therapy, makes it a desirable treatment modality for the management of various bacterial infections, including those caused by resistant pathogens such as MRSA and QRSA. Features of levonadifloxacin, such as availability of both IV and oral form, minimal drug-drug interactions, lack of the need to adjust dosages in renal and hepatically impaired patients along with a broad spectrum of coverage, make it a suitable agent that meets several unmet clinical needs of physicians.
